# Supplementary figures and images for: Genome-wide conditional degron libraries for functional genomics
Source: J Cell Biol. 2024 Dec 18;224(2):e202409007. doi: 10.1083/jcb.202409007 (PMC11654235; doi:10.1083/jcb.202409007)

$\alpha$ -myc antibody

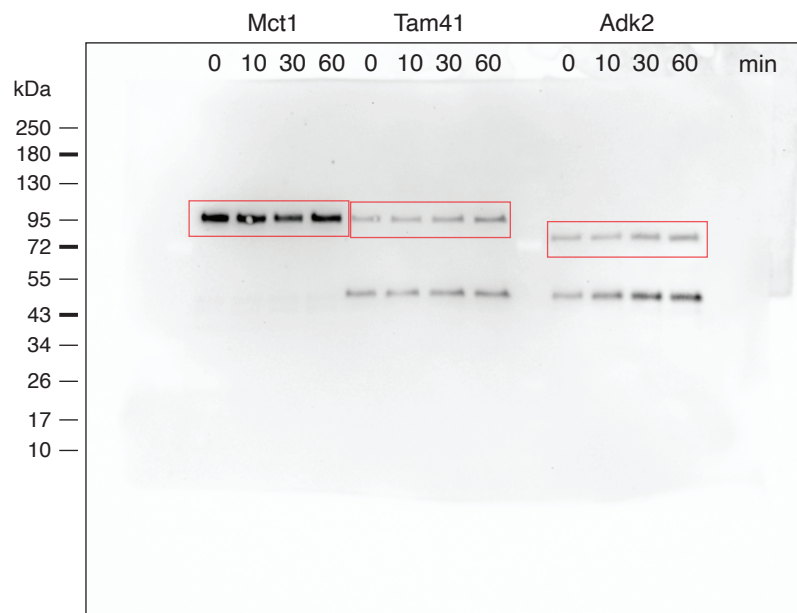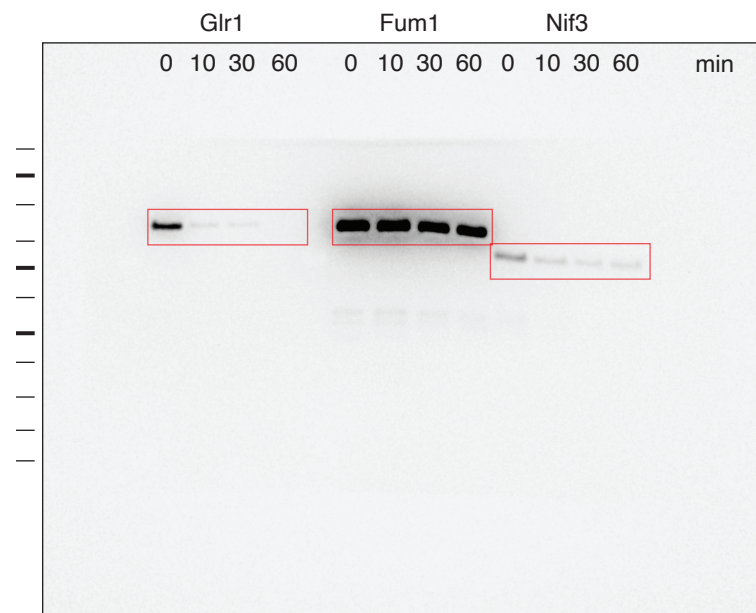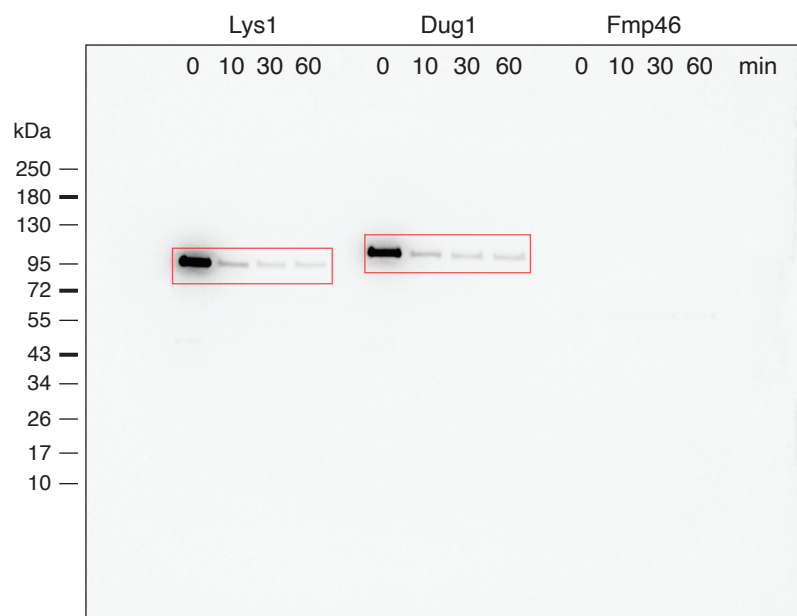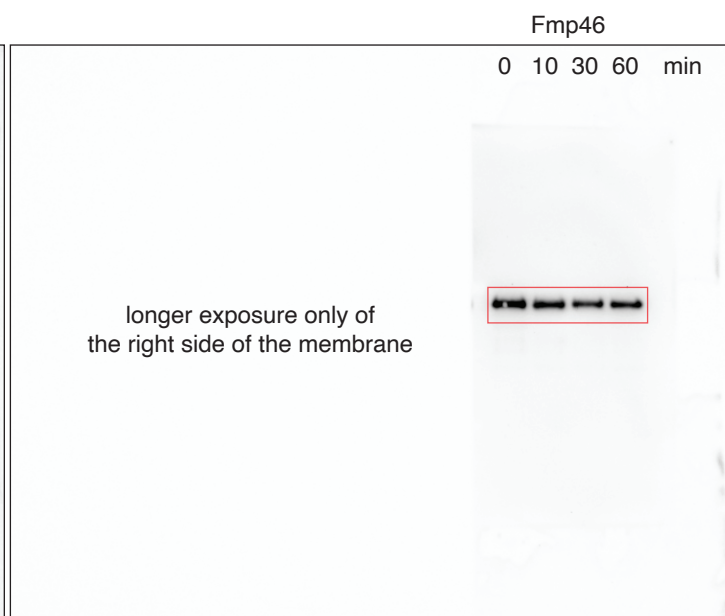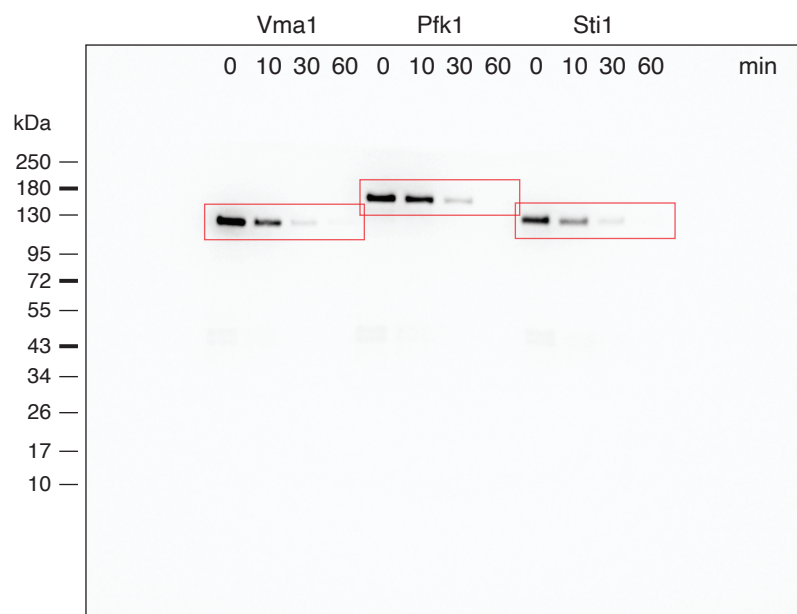

α-Pgk1 antibody

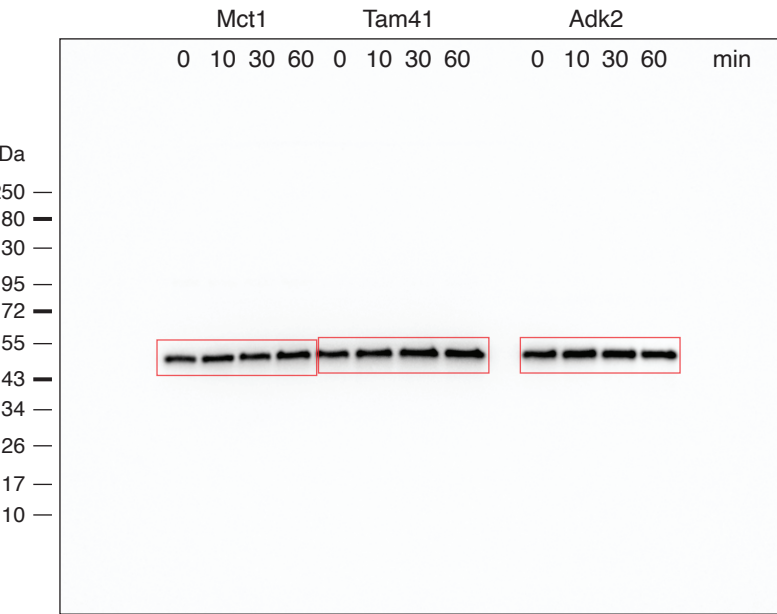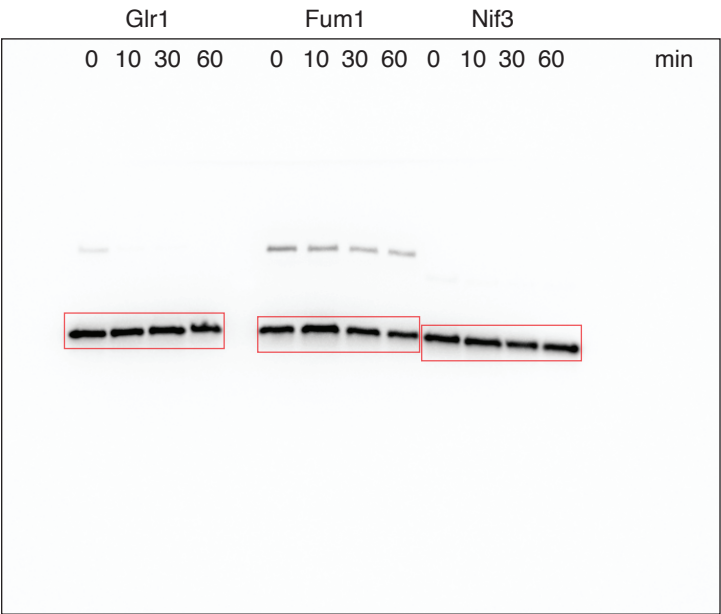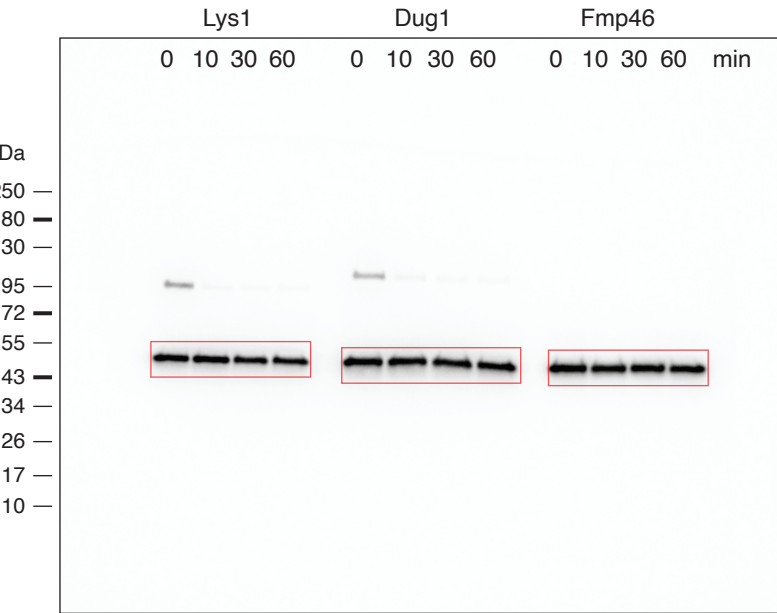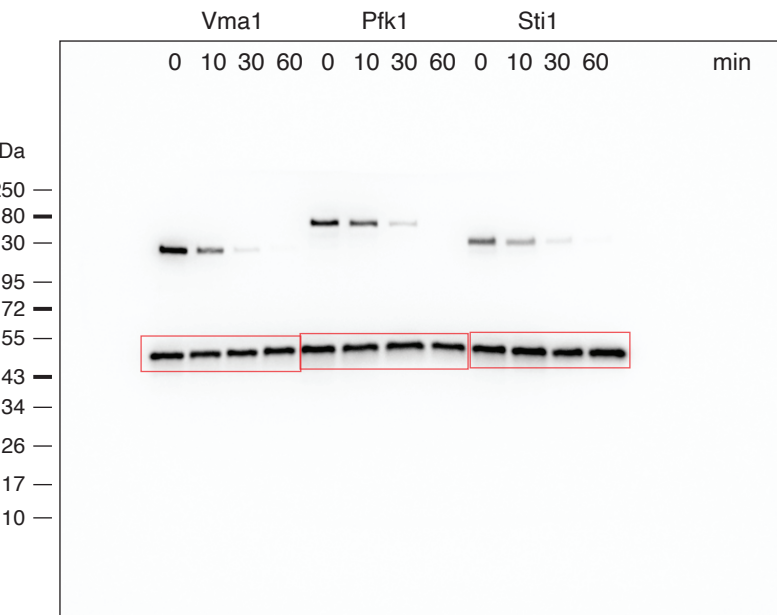

Ponceau

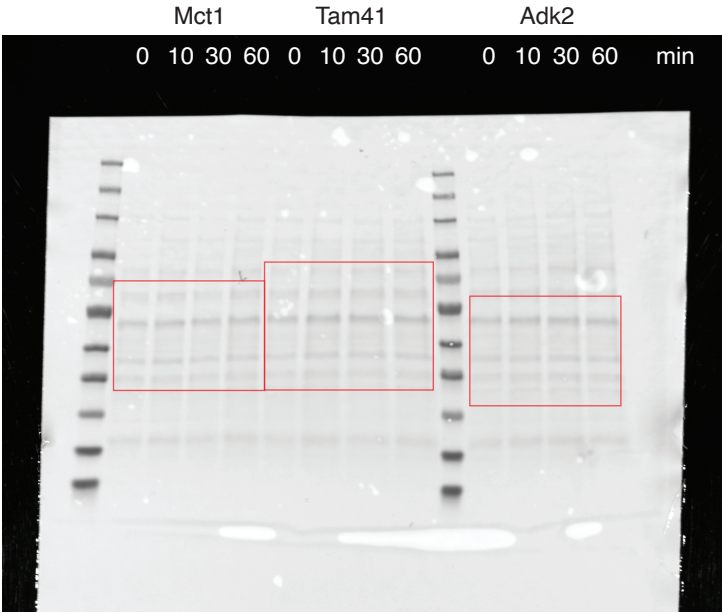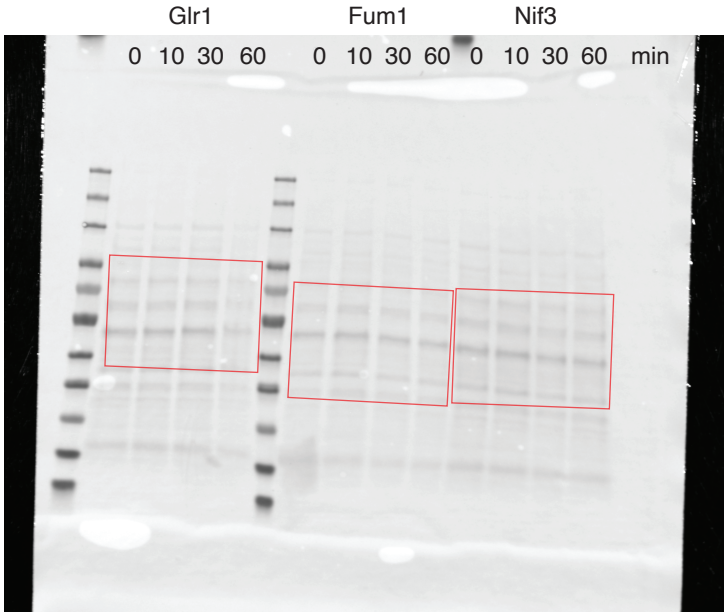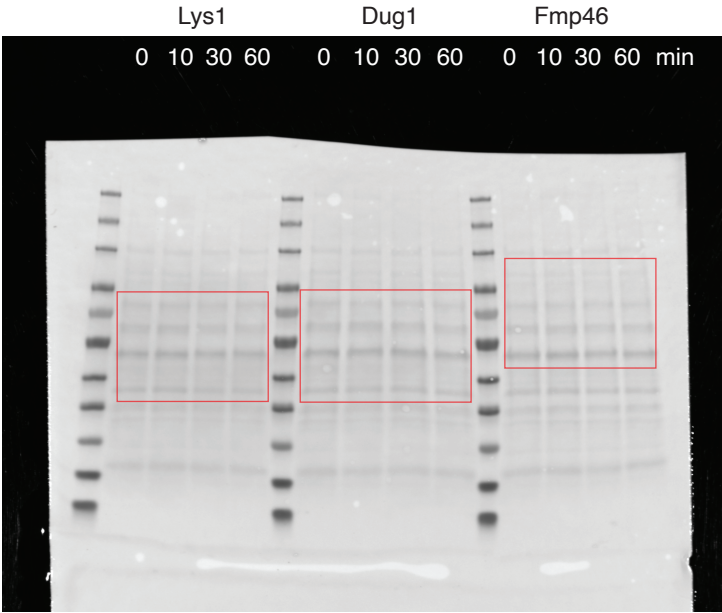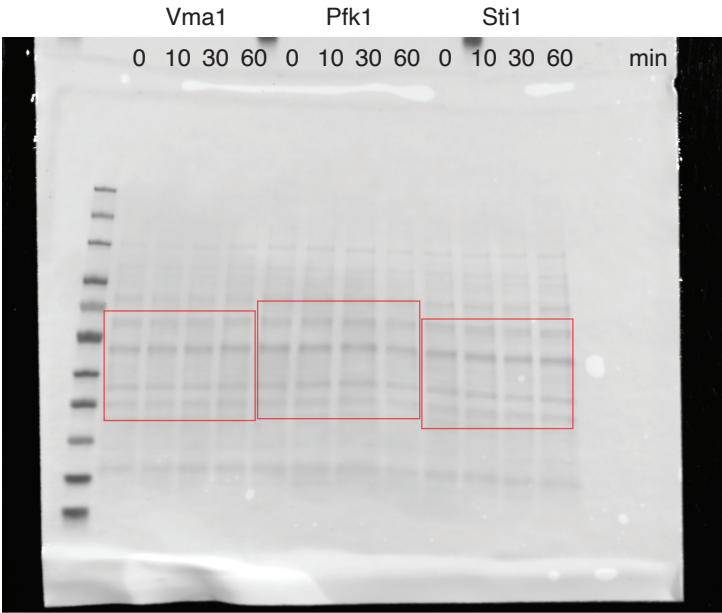

Supplement: SourceData FS2 — is the source file for Fig. S2. [file jcb_202409007_sourcedatafs2.pdf]
